# Breast MRI: does a clinical decision algorithm outweigh reader experience?

**DOI:** 10.1007/s00330-022-09015-8

**Published:** 2022-07-19

**Authors:** Nina Pötsch, Aida Korajac, Philipp Stelzer, Panagiotis Kapetas, Ruxandra-Iulia Milos, Matthias Dietzel, Thomas H. Helbich, Paola Clauser, Pascal A. T. Baltzer

**Affiliations:** 1 Division of General and Pediatric Radiology, Department of Biomedical Imaging and Image-guided Therapy, Medical University of Vienna and General Hospital, Waehringer Guertel 18-20, A-1090 Vienna, Austria; 2 Institute of Radiology, Erlangen University Hospital, Maximiliansplatz 2, 91054 Erlangen, Germany

**Keywords:** Algorithms, MRI, BI-RADS, Breast neoplasms, Radiology

## Abstract

**Objectives:**

Due to its high sensitivity, DCE MRI of the breast (MRIb) is increasingly used for both screening and assessment purposes. The Kaiser score (KS) is a clinical decision algorithm, which formalizes and guides diagnosis in breast MRI and is expected to compensate for lesser reader experience. The aim was to evaluate the diagnostic performance of untrained residents using the KS compared to off-site radiologists experienced in breast imaging using only MR BI-RADS.

**Methods:**

Three off-site, board-certified radiologists, experienced in breast imaging, interpreted MRIb according to the MR BI-RADS scale. The same studies were read by three residents in radiology without prior training in breast imaging using the KS. All readers were blinded to clinical information. Histology was used as the gold standard. Statistical analysis was conducted by comparing the AUC of the ROC curves.

**Results:**

A total of 80 women (median age 52 years) with 93 lesions (32 benign, 61 malignant) were included. The individual within-group performance of the three expert readers (AUC 0.723–0.742) as well as the three residents was equal (AUC 0.842–0.928), *p* > 0.05, respectively. But, the rating of each resident using the KS significantly outperformed the experts’ ratings using the MR BI-RADS scale (*p* ≤ 0.05).

**Conclusion:**

The KS helped residents to achieve better results in reaching correct diagnoses than experienced radiologists empirically assigning MR BI-RADS categories in a clinical “problem solving MRI” setting. These results support that reporting breast MRI benefits more from using a diagnostic algorithm rather than expert experience.

**Key Points:**

• *Reporting breast MRI benefits more from using a diagnostic algorithm rather than expert experience in a clinical “problem solving MRI” setting*.

• *The Kaiser score, which provides a clinical decision algorithm for structured reporting, helps residents to reach an expert level in breast MRI reporting and to even outperform experienced radiologists using MR BI-RADS without further formal guidance*.

**Supplementary Information:**

The online version contains supplementary material available at 10.1007/s00330-022-09015-8.

## Introduction

Contrast-enhanced magnetic resonance imaging of the breast (MRIb) is well established in breast imaging for various indications. Standard applications include screening in high-risk patients, diagnostic work-up of equivocal lesions in mammography and ultrasound, or treatment planning and monitoring in breast cancer patients [1, 2]. Moreover, recent data are promising regarding MRIb for intermediate risk screening in women with extremely dense breasts [3–5]. Therefore, an increase in the demand for MRIb examinations is envisioned in the near future.

Interpretation of MRIb is considered a task for well-trained radiologists. Since the interpretation of MRIb is challenging and inter-reader agreement is variable [6, 7], improvement in standardized image interpretation is an active research topic [1, 8–14]. The Breast Imaging Reporting and Data System (BI-RADS) lexicon of the American College of Radiology (ACR) in the current 5^th^ edition undoubtedly serves as the most widely accepted standard for reporting MRIb findings [10]. Nevertheless, inter-observer variability and diagnostic accuracy vary using the standardized BI-RADS lexicon [15–19]. This may be explained by the fact that the BI-RADS lexicon does not include a clinical decision algorithm that formally combines diagnostic criteria for structured reporting of MRIb [20].

The Kaiser score (KS) provides such clinical decision rules that uses machine learning methodology to combine five independent MR BI-RADS criteria: (i) spiculations; (ii) SI-time curve type; (iii) margins of the lesion; (iv) internal enhancement; and (v) presence of edema. These criteria are weighted differently in their combination and result in a score from 1 “lowest risk of breast cancer” to 11 “highest risk of breast cancer.” This score can then be directly translated into a BI-RADS category rating (KS 1–4 = BI-RADS 2/3, KS 5–7 = BI-RADS 4, KS 8–11 = BI-RADS 5) with corresponding clinical implications [21]. Thereby, the KS has shown to facilitate the characterization of enhancing breast lesions, potentially avoiding misdiagnosis and unnecessary biopsies [22–24]. In addition, initial results have suggested that the KS compensates for reader experience and improves inter-reader agreement [25]. To investigate the influence of reader experience, however, independent, off-site readers experienced in breast MRI, but not previously exposed to the KS, should be compared to readers inexperienced in breast MRI who simply apply the KS as a diagnostic algorithm in the same patients.

Therefore, the aim of our study was to evaluate the diagnostic performance of residents using the KS for structured MRIb reporting compared to radiologists with experience in breast imaging using only MR BI-RADS.

## Methods

### Study design

The local ethics committee approved this retrospective, single-center study, waiving the need for informed consent (IS 1917/2016). The patient-related data were de-identified and handled in accordance with standards of good scientific practice. All data generated and analyzed during this study are available from the corresponding author by request. The same dataset has already been described in a previous analysis with a completely different study design and aims [26].

### Patients

We included a subset of patients, prospectively recruited in the context of another study [26]. The inclusion criteria were defined as follows: female patients > 21 years undergoing MRIb for inconclusive or suspicious findings (BI-RADS 0, 4, and 5) on conventional imaging (mammography/tomosynthesis, ultrasound, clinical examination) [27]. Pregnant or lactating women, as well as women with breast implants or a history of breast cancer and related treatment, were excluded. Moreover, women unable to give written, informed consent or with contraindications for MRI or gadolinium-based contrast agents were excluded. Histopathology, acquired through stereotactic, US-, or MRI-guided biopsy, was used as the standard of reference. Patients not undergoing biopsy, due to distinct benign MRI findings that did not require biopsy, were excluded from further analysis.

### MRI acquisition

The MRIb studies included were mainly performed at our institution (*n* = 48, 60%) or at outside facilities (*n* = 32, 40%) using either 1.5-T or 3-T scanners, with dedicated eight-channel (at least) breast coils. All images were acquired with the patient in a prone position using a standard protocol in line with European Society of Breast Imaging (EUSOBI) recommendations [27], including, at least, a T2-weighted sequence and a gradient echo T1-weighted sequence, prior to and after i.v. injection of a gadolinium-based contrast agent, in the axial plane. A minimum of three T1-weighted post-contrast sequences was acquired. For improved evaluation of contrast enhancement, subtracted images were available. Details on the acquired series are given as Supplemental Material (Supplemental Table 1).

### Image interpretation

Image interpretation was performed by three, independent, off-site radiologists with more than 15 years of experience in MRIb. Comparable to clinical routine, the expert readers rated the following: (i) presence and location of a lesion (breast quadrant); (ii) lesion type (mass or non-mass enhancement); and (iii) lesion size (mm); and assigned an MR BI-RADS [10] score for each lesion. The readers were instructed to rate only the most suspicious lesion (*n* = 1) per breast.

The same studies were analyzed independently by three, off-site radiology residents using the Kaiser score (KS), prior to their basic (ESR level I) rotation in “breast radiology,” with little to no experience in MRIb. Briefly, the KS is a simple classification system based on a total of five kinetic and morphologic criteria (I: contrast enhancement kinetics, II: internal enhancement, III: presence or absence of spiculations, IV: lesion margins, and V: edema) resulting in assignment categories from 1 (= lowest risk of breast cancer) to 11 (= highest risk of breast cancer). Generally, a KS rating higher than 4 corresponds to a BI-RADS 4/5 and is, therefore, considered suspicious with a consequent indication for biopsy [21]. The residents used the publicly available KS online tool (accessible via: http://www.meduniwien.ac.at/kaiser-score/), which queries the individual criteria one by one and automatically provides a KS rating and the corresponding BI-RADS category. Residents were aware of the lesion’s location (side and quadrant) and size, but were blinded to the experts’ MR BI-RADS rating to ensure the exact same lesion was rated by experts and trainees. All readers were blinded to all clinical information and patient history. No prior imaging studies were available to the readers.

### Statistical analysis

Statistical analysis was done using SPSS 22 for MAC (IBM). Relevant clinical and demographic and data are presented by descriptive analyses. Metric continuous data are given as median and ranges. The individual diagnostic performance differentiating benign and malignant breast lesion was assessed by comparing the AUC of the ROC curves. Inter-reader agreement for expert and resident readers was evaluated using Cohen’s kappa. A two-sided *p* value of *p* < 0.05 was considered significant.

## Results

A total of 80 women (median age 52 years, range 34–83 years) with 93 lesions, 32 benign and 61 malignant, were included. Twenty lesions were rated as non-mass lesions, 13 of which were malignant. Median lesion size was 18 mm (range 4–80 mm). Histologic details are given in Table 1.

**Table 1:** Histologic details of the 93 lesions included in this analysis

Histology	Number (%)
Benign	32
Fibrocystic changes	15 (47%)
Papilloma	6 (19%)
Inflammatory changes	6 (19%)
Fibroadenoma	5 (15%)
Malignant	61
Invasive carcinoma NST	19 (31%)
Invasive lobular carcinoma	2 (3)
Invasive carcinoma with DCIS	25 (41%)
DCIS	15 (25%)

*NST*, nonspecific type; *DCIS*, ductal carcinoma in situ

**Fig. 1:**
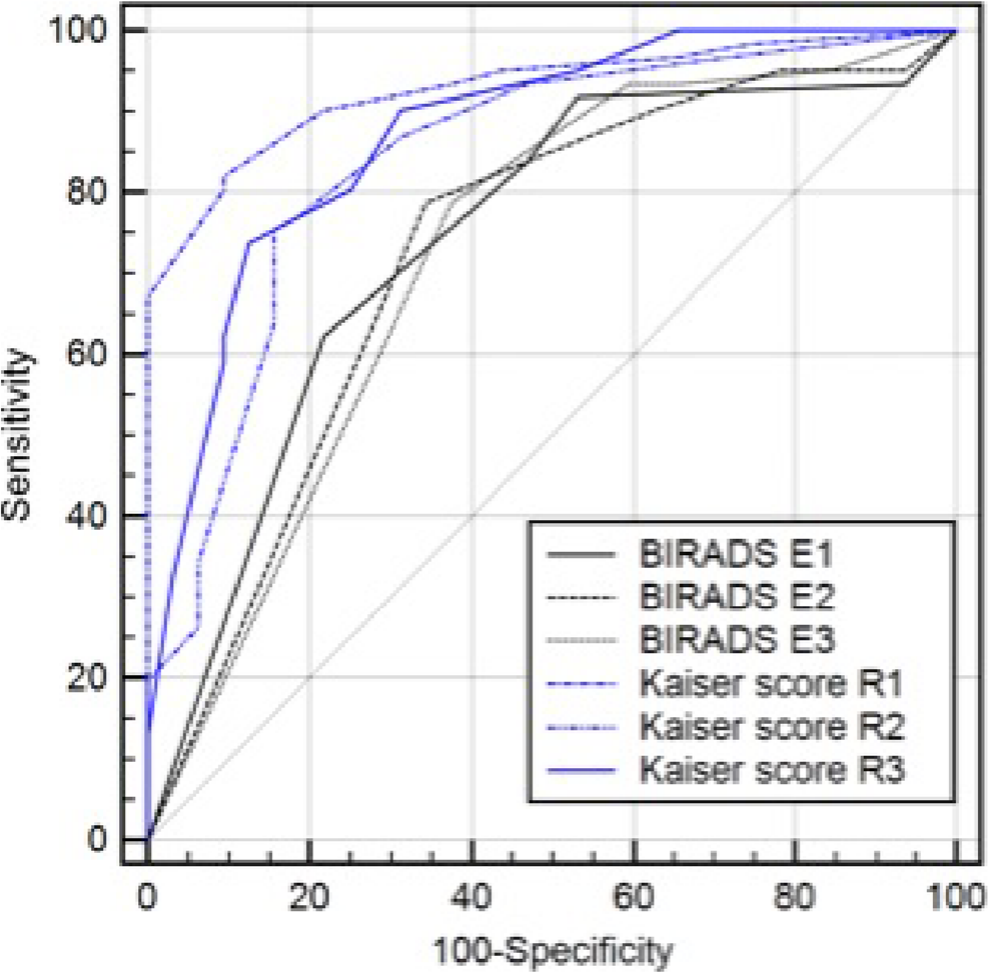
ROC curves demonstrating the performance of the residents (depicted in blue) and of the experts (depicted in black)

**Fig. 2:**
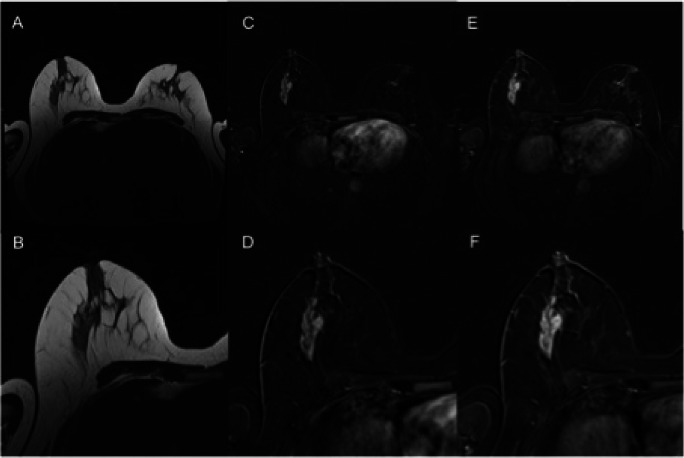
B2 lesion rated BI-RADS 4/5 by experts and benign using the KS. T2 TSE (**A**), early (**C**) and late (**E**) post-contrast T1 with fat suppression, as well as corresponding magnifications of the right breast (**B**, **D**, **F**). This lesion was rated as MR BI-RADS 4 or 5 by the experts. Applying the Kaiser score to this mass lesion without spiculations, persistent enhancement, and irregular margins gives a Kaiser score of 3, which is a benign result corresponding to a BI-RADS 2/3. Histologic verification showed tumor-free mammary parenchyma with low-grade pseudoangiomatous stromal changes, B2

**Fig. 3:**
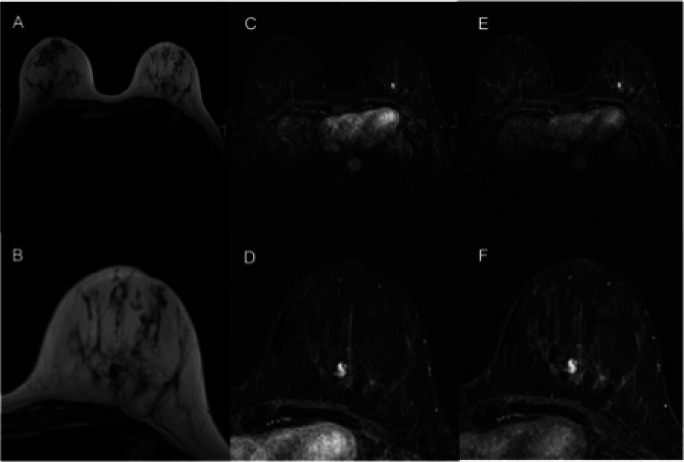
Fibroadenoma rated BI-RADS 4/5 by experts and benign using the KS. T2 TSE (**A**), early (**C**) and late (**E**) post-contrast T1 with fat suppression, as well as corresponding magnifications of the left breast (**B**, **D**, **F**). This lesion was rated as MR BI-RADS 4 or 5 by the experts. Applying the Kaiser score to this mass lesion without spiculations, plateau enhancement, and circumscribed margins gives a Kaiser score of 2, which is a benign result corresponding to a BI-RADS 2/3. Histologic verification showed a fibroadenoma, B2

**Fig. 4:**
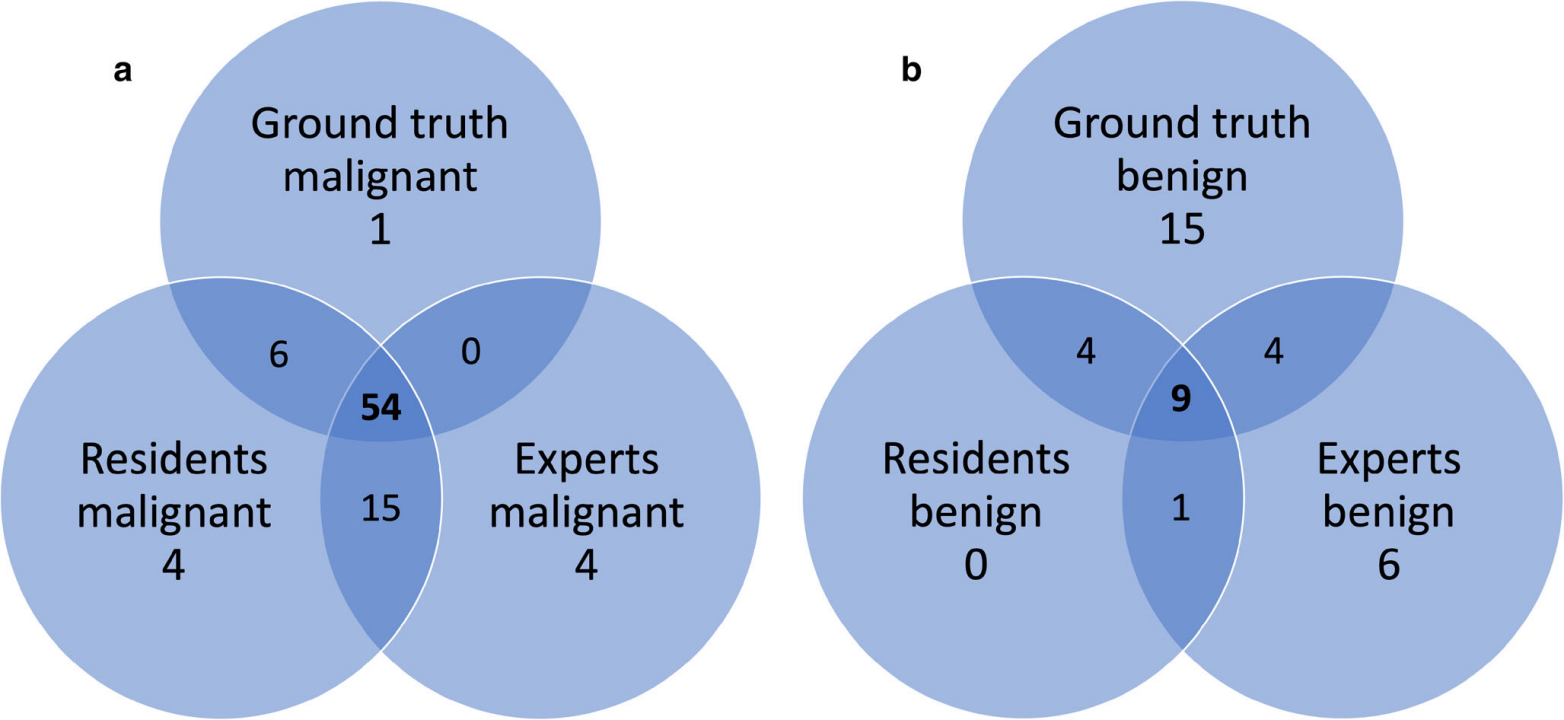
Venn diagram depicting concordant findings in expert and resident readers together with histologic ground truth for (**a**) malignant lesions and (**b**) benign lesions. For the Kaiser score, an exploratory cut-off was set at a value of 3. Thus, lesions with a rating < 3 were considered benign and lesions with a rating ≥ 3 were considered malignant

Using MR BI-RADS only, the performance between the three expert readers (AUC 0.723–0.742, *p* > 0.72) did not differ significantly. Using the KS, the difference in the performance between the three residents (AUC 0.842–0.928, *p* = 0.03, *p* > 0.08, respectively; Table 2) varied between significance and borderline significance. The rating of each single resident using the KS significantly outperformed the experts’ ratings using the MR BI-RADS scale (*p* < 0.01–0.05; Fig. 1). Moreover, we found a tendency toward better inter-reader agreement using the KS for structured reporting compared to MR BI-RADS (KS: kappa 0.579–0.710, MR BI-RADS: kappa 0.531–0.624). Exemplary images from the study population are shown in Figs. 2 and 3. Figure 4 shows concordant findings in expert and resident readers depicted against ground truth.

**Table 2:** AUC values for each individual reader

	AUC	95% CI
Expert 1	0.742	0.641–0.827
Expert 2	0.727	0.625–0.814
Expert 3	0.723	0.621–0.811
Resident 1	0.928	0.856–0.971
Resident 2	0.842	0.752–0.909
Resident 3	0.875	0.790–0.935

## Discussion

Our study shows that the KS, which provides a clinical decision algorithm for structured reporting, helps residents to reach an expert level in MRIb reporting. Residents who applied the KS for MRIb reading achieved even better results than expert readers using only MR BI-RADS. Moreover, there was also a tendency for better inter-reader agreement using the KS. These results support that successful structured reporting of MRIb using the KS does not require expert training.

Prior studies have demonstrated and validated the diagnostic value of the KS in several clinical MRIb scenarios [22–25, 28–30]. It is applicable in high-risk, intermediate-risk, and average-risk patients alike; can be used for breast cancer diagnosis independent of mammographic appearance; and has a substantial potential to reduce unnecessary biopsies in a broad variety of indications [22–25, 28, 29]. The conceivable impact on inexperienced readers has been suggested previously [22, 25]. Marino et al demonstrated better reader performance for a less experienced reader in an intra-individual comparison study using MR BI-RADS with and without the KS. Overall, they reported that using empirical MR BI-RADS interpretation led to significant differences between readers based on their individual level of experience, while using the KS did not. Our findings go beyond this, as they do not only corroborate the suggestion that the application of the KS could compensate for reader experience, but show that inexperienced readers outperform even experts who did not use algorithmic guidance for structured reporting. The independent reader approach used in this study is more valid, as the prior study allowed the same readers to use the KS after the initial read without any attempt to alleviate a possible recognition bias by a randomized reading approach. In addition, the readers were trained in using the KS before the beginning of the study, and thus, may have subconsciously been influenced by the KS. Our approach avoided such a potential influence and the experienced readers were neither specifically exposed to nor did they apply the KS in their routine clinical practice. And, our results show that MRIb interpretation, relying on the KS as a decision algorithm, seemingly does not require expert training. This is important considering the increasing demand on MRIb examinations because of the medical and economic evidence in favor of extended MRIb indications [3, 31–36].

Image interpretation in MRIb is considered a task for experienced and specifically trained radiologists [9]. Why? The main reason is complexity: a number of different parameters in various MRI sequences have to be considered and need to be combined in a final BI-RADS rating, which is not based on a formal decision rule, but—rather annoyingly put—on gut feeling. In addition, the physiological background enhancement, which varies inter-individually, might lead to diagnostic difficulties [37]. These factors might contribute to the variability of inter-reader agreement in MRIb image interpretation [6, 7]. For instance, Grimm et al [17] described only a limited inter-reader agreement between experts who reviewed BI-RADS 3 lesions using the current 5^th^ edition of the MR BI-RADS lexicon [10]. Complexity and lack of formal guidance for structured reporting within the diagnostic process, with an unclear weighting of the individual criteria, may also explain why some authors describe comparable diagnostic performance metrics between MRIb with full compared to abbreviated protocols [38]. The KS, as a clinical decision rule based on predefined and statistically weighted MR BI-RADS criteria, does address these needs [22]. In line with this reasoning, several studies have demonstrated that if the Kaiser score as a clinical decision rule is used for breast MRI interpretation, the additional value of DWI is negligible [24, 30, 39].

Our study has some limitations, which should be addressed. First of all, initial data were collected in the framework of another study, including only patients with a final BI-RADS category 4 or 5 assessment who received invasive management [26]. These selection criteria imply a study population enriched with clinically challenging cases. This resulted in a correspondingly higher proportion of carcinomas, leading to a possible overestimation of sensitivity and underestimation of specificity. Therefore, the results strictly apply to the investigated setting, and the impact of the KS may be less pronounced in, e.g., a screening population. In addition, the inexperienced readers classified only indicated lesions and did not perform a detection task. Even though lesion detection in breast MRI is not considered a problem in the literature, that study did not answer whether high experience is required to identify lesions upfront [8, 20, 38]. However, the results are rather striking, as all the aforementioned potential biases would instead be in favor of a higher diagnostic performance for the experienced radiologists.

In conclusion, structured MRIb reporting, using the KS to reach an objective diagnostic category, helped residents to achieve better results than experienced radiologists who empirically assigned MR BI-RADS categories in a clinical “problem solving MRI” setting. These results support that reporting breast MRI benefits more from using a diagnostic algorithm rather than expert experience.

## Supplementary information

ESM 1 Supplemental Figure 1: Study flow chart of patients included and excluded. Supplemental Table 1: Sequences and vendors of the MRI examinations included. a View-sharing, 3D, time-resolved angiography with stochastic trajectory, gradient echo sequence; b 3D fast low angle shot T1 Dixon sequence; c 3D fast low angle shot anisotropic T1-weighted sequence without fat saturation; d Gradient echo 3D without fat suppression (PDF 95 kb)

## Abbreviations

ACR: American College of Radiology
BI-RADS: Breast Imaging Reporting and Data System
EUSOBI: European Society of Breast Imaging
KS: Kaiser score
MRIb: Breast MRI

## Declarations

### Guarantor

The scientific guarantor of this publication is Pascal A.T. Baltzer.

### Statistics and biometry

One of the authors has significant statistical expertise. No complex statistical methods were necessary for this paper.

### Study subjects or cohorts overlap

Some study subjects or cohorts have been previously reported in Clauser et al *Magn. Reson. Imaging JMRI*. 2020;52(2):589–595.

### Methodology

• retrospective

• diagnostic study

• multicenter study
