# Supplementary material for: Breast MRI: does a clinical decision algorithm outweigh reader experience?
Source: Eur Radiol. 2022 Jul 19;32(10):6557–64. doi: 10.1007/s00330-022-09015-8 (PMC9474540; doi:10.1007/s00330-022-09015-8)

Supplemental Table 1. Sequences and vendors of the MRI examinations included.

**Vendor**	Siemens	Siemens	Siemens	Philips
**Tesla**	3	1.5	1.5	1.5
**T1- weighted sequences**				
**Sequence**	TWIST [a]	fl3D Dixon [b]	fl3D dynamic [c]	T1 FFE3D [d]
**TR**	6.23	10	8.9	7.05
**TE**	2.95	2.39	4.7	4.6
**Spatial resolution**	0,9x0,9x2	0,7x0,7x2	0,8x0,8x2	1x1x2
**Slices**	144	80	140	132
**Temporal resolution**	28s, one pre- and 10 post-contrast	70s, one pre- and 3 post-contrast	110s, one pre- and 5 post-contrast	72s, one pre- and 3 post-contrast
**T2- weighted sequences**				
**Sequence**	T2-TSE	T2-TSE	TIRM Blade	eSTIR
**TR**	4630	3990	6990	4039.91
**TE**	194	183	121	65
**TI**	/	/	160	175
**Spatial resolution**	0,7x0,7x3	0,7x0,7x3	0,8x0,9x4	0,7x0,7x4
**Slices**	65	48	32	50

[a] View-sharing, 3D, time-resolved angiography with stochastic trajectory, gradient echo sequence; [b] 3D fast low angle shot T1 Dixon sequence; [c] 3D fast low angle shot anisotropic T1-weighted sequence without fat saturation; [d] Gradient echo 3D without

fat suppression

Supplemental Figure 1: Study flow chart of patients included and excluded

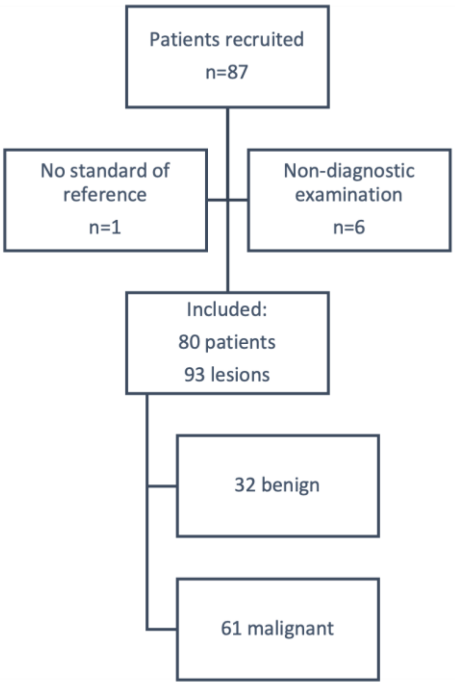

Supplement: Supplementary file 1 — Supplemental Figure 1: Study flow chart of patients included and excluded. Supplemental Table 1: Sequences and vendors of the MRI examinations included. a View-sharing, 3D, time-resolved angiography with stochastic trajectory, gradient echo sequence; b 3D fast low angle shot T1 Dixon sequence; c 3D fast low angle shot anisotropic T1-weighted sequence without fat saturation; d Gradient echo 3D without fat suppression (PDF 95 kb) [file 330_2022_9015_MOESM1_ESM.pdf]
